# Tracking of Bacteriophage Predation on *Pseudomonas aeruginosa* Using a New Radiofrequency Biofilm Sensor

**DOI:** 10.3390/s24072042

**Published:** 2024-03-22

**Authors:** Matthieu Longo, Florian Lelchat, Violette Le Baut, Stéphane Rioual, Fabienne Faÿ, Benoit Lescop, Claire Hellio

**Affiliations:** 1 Univ Brest, Lab-STICC, CNRS, UMR 6285, F-29200 Brest, France; longo@cervval.com (M.L.); rioual@univ-brest.fr (S.R.); 2 Univ Brest, BIODIMAR/LEMAR, CNRS, UMR 6539, F-29200 Brest, France; claire.hellio@univ-brest.fr; 3 Leo Viridis, 245 Rue René Descartes, F-29280 Plouzané, France; lelchat@leoviridis.fr (F.L.); le-baut@leoviridis.fr (V.L.B.); 4 Laboratoire de Biotechnologie et Chimie Marines, Centre de Recherche Saint Maudé, Université Européenne de Bretagne, Université de Bretagne-Sud, F-56321 Lorient, France; fabienne.fay@univ-ubs.fr

**Keywords:** bacteriophage, biofilm, *Pseudomonas aeruginosa*, radiofrequency characterization, sensor

## Abstract

Confronting the challenge of biofilm resistance and widespread antimicrobial resistance (AMR), this study emphasizes the need for innovative monitoring methods and explores the potential of bacteriophages against bacterial biofilms. Traditional methods, like optical density (OD) measurements and confocal microscopy, crucial in studying biofilm–virus interactions, often lack real-time monitoring and early detection capabilities, especially for biofilm formation and low bacterial concentrations. Addressing these gaps, we developed a new real-time, label-free radiofrequency sensor for monitoring bacteria and biofilm growth. The sensor, an open-ended coaxial probe, offers enhanced monitoring of bacterial development stages. Tested on a biological model of bacteria and bacteriophages, our results indicate the limitations of traditional OD measurements, influenced by factors like sedimented cell fragments and biofilm formation on well walls. While confocal microscopy provides detailed 3D biofilm architecture, its real-time monitoring application is limited. Our novel approach using radio frequency measurements (300 MHz) overcomes these shortcomings. It facilitates a finer analysis of the dynamic interaction between bacterial populations and phages, detecting real-time subtle changes. This method reveals distinct phases and breakpoints in biofilm formation and virion interaction not captured by conventional techniques. This study underscores the sensor’s potential in detecting irregular viral activity and assessing the efficacy of anti-biofilm treatments, contributing significantly to the understanding of biofilm dynamics. This research is vital in developing effective monitoring tools, guiding therapeutic strategies, and combating AMR.

## 1. Introduction

Bacterial biofilms, which are complex and dynamic structures, provide significant ecological benefits to micro-organisms, including nutrient supply, enhanced survival rates, and resistance to harsh conditions and biocides [1]. These advantages are particularly pronounced under stress, making biofilms a crucial strategy for bacterial persistence. However, biofilm formation poses substantial challenges across various industries, notably aquaculture, energy, and shipping, due to its role in mechanical failures and chronic contamination [2]. The growing resistance of biofilms to traditional antimicrobial agents underscores the urgency for alternative strategies and efficient monitoring methods [3,4], notably, in the context of the generalized antimicrobial resistance (AMR) outbreak [5] where fast monitoring of contamination is often an asset [6].

Recent research has highlighted bacteriophages as promising agents against bacterial biofilms. Their ability to disrupt biofilms through specific enzymes and adapt to bacterial resistance mechanisms represents a significant advancement [7,8,9,10,11,12]. Conventional approaches to study biofilm–virus interactions include colony count (CFU/mL) of sessile cells, optical density assessments, and colorimetric assays to quantify biomass and interactions within the biofilm [13,14]. Furthermore, visualization techniques, such as Transmission Electron Microscopy (TEM), Scanning Electron Microscopy (SEM), Atomic Force Microscopy (AFM), and confocal microscopy, provide detailed insights into biofilm development and phage therapy effects [15,16]. However, these methods are often costly, time intensive, and limited in their ability to monitor long-term biofilm growth dynamics.

Despite their usefulness, these traditional methods have notable limitations. For example, culture-based techniques do not capture the full diversity of microbial communities in biofilms, and microscopic methods, while offering precise details of biofilm structure, are generally laborious and unsuitable for real-time monitoring. In addition, most of these techniques do not allow rapid detection and are not always sensitive enough to detect the early stages of biofilm formation or low concentrations of pathogenic bacteria.

In recent years, numerous research efforts have been made to develop low-cost, highly sensitive, and fast biosensors to overcome these limitations. For example, Brunetti et al. [17] worked on a novel micro-nano optoelectronic biosensor for label-free real-time biofilm monitoring and Therisod et al. [18] presented significant advances in biofilm monitoring and bacterial type differentiation. The use of resonant hyperspectral imaging has also shown progress in studying the antibiotic response of biofilms at an early stage [19]. In addition, innovative platforms, such as the DEP on-chip device for the precise sorting of live/dead bacteria and impedimetric immunosensors for the detection of pathogens and biomarkers, offer promising prospects for the diagnosis and management of AMR [20,21]. In this context, the development of our radio frequency sensor based on dielectric permittivity measurements of biofilms opens up new prospects for detecting and monitoring their appearance and dynamics in an immersed environment. This sensor aims to fill the gaps left by conventional methods by offering a non-invasive, cost-effective, and efficient solution for real-time biofilm monitoring. This could prove crucial in guiding antibiotic choice and effective biofilm removal, thus contributing to the fight against antimicrobial resistance.

Our current study builds on our previous work [22], where we developed a sensor for real-time bacterial growth monitoring based on radio frequency measurements of biological electrical properties. This paper focuses on the potential applications of the biofilm sensor in monitoring biofilm and bacterial growth, evaluating its effectiveness compared to traditional optical density measurements and microscopy. We used *Pseudomonas aeruginosa* PAO1, a biofilm-producing bacteria considered to be one of the top four critical opportunistic pathogens for humans by the World Health Organization (WHO), as a model micro-organism [23]. Since the end of the last century, this strain is often involved in nosocomial diseases [24], which will represent an estimated annual loss of 0.14% of global Gross Domestic Product (GDP) in the very near future [25]. Most importantly, AMR infection affected the health of 47.9 million persons and directly caused 1270 annual estimated deaths in 2019 [26]. This late number was much more important when the infection was a copathology with an estimated death toll of 1.170 million [27]. The cardinal reason for such virulence is the vast antibiotics resistance mechanism exhibited by Multidrug-Resistant Bacteria (MRB), such as *Pseudomonas aeruginosa* PAO1, which could dramatically decrease life prognosis for patients suffering from comorbidity factors (e.g., cystic fibrosis, burn victims, radio-induced aplasia, immunocompromised transplanted patients, elders) [28]. Considering *Pseudomonas aeruginosa* PAO1, this antibioresistance can be increased by its ability to switch from a planktonic state to a biofilm way of life [29]. This biofilm is generally made of alginate exopolysaccharide, a biopolymer of complex acetylated mannuronic acid residue chains [29]. This biofilm has multiple roles for the bacteria, such as protecting the strain from toxic compounds from the surrounding environment, including antibiotics [30]. Once established in biofilm, the eradication of the bacteria has been proven to be very problematic and fastidious [31]. For a good prevention/eradication strategy, it is thus crucial to intervene at the first steps of biofilm formation, even using powerful tools such as wild or engineered bacteriophages [32,33]. The biofilm has several growth phases [34,35], and substantial levels of EPS (exopolysaccharides) will be produced. Our study demonstrates that this sensor is suitable for detecting these growth phases effectively. We tested this sensor using a culture model of *Pseudomonas aeruginosa* strain PAO1 and the lytic phage Domino-17, aiming to validate its utility as a real-time biofilm monitoring tool.

## 2. Materials and Methods

### 2.1. Biological Material (Micro-Organisms, Preculture, and Cell Growth)

The strain *Pseudomonas aeruginosa* PAO1 is used for the experiments as a model species for biofilm formation. Aliquots of bacterial cultures are stored frozen at −80 °C in a glycerol medium (10%). Prior to the experimental work, the strain is inoculated onto M1 agar medium (tryptone—10 g/L; meat extract—5 g/L; sodium chloride—5 g/L) and then incubated at 37 °C for 24 h, which is the optimum temperature for the biofilm production with this strain. The culture obtained is then used for the different tests.

Domino-17 bacteriophages (Pseu**DOM**onas aerug**IN**osa Pa**O**1 virus 17) were previously isolated from wastewater amplified and characterized by Leo viridis. A spot test is performed by inoculating *P. aeruginosa* into an M1 agar medium following the protocol of Lelchat et al. [36] with the aim of isolating viral strains presenting a strong lytic cycle. When the medium is sufficiently solidified, 5 µL of virus solution is deposited in the center of the test plate. The whole assembly is incubated at 37 °C. After 24 h of incubation, the appearance of a lysis plaque reveals the presence of a lytic bacteriophage. The halo, visible in the center of the plate, means that the viruses are depolymerizing the matrix of the bacterial biofilm and destroying the cells. Longer incubation time will result in a halo that will enlarge considerably. To amplify the virus in large quantities, a lysis plaque is extracted and resuspended in an SM buffer, inoculated in an M1 medium in the presence of the host, and incubated at 37 °C under agitation (50 rpm) for at least one night. The virus is then recovered by centrifuging the culture at 11,000 rpm and filtering the supernatant at 0.2 µm.

### 2.2. Optical Density Assessment and Growth Monitoring

Bacterial growth was monitored by automatically reading the optical density (at 600 nm) of bacterial solutions every 10 min for 30 h using a TECAN InfiniteTM spectrophotometer according to the protocol described in the work by Lelchat et al. [37]. Measurements were performed in a polystyrene plate with 6 wells, each representing a replica. Each well containing bacterial inoculum was incubated at 37 °C, and then virions were injected into solution after the first 6 h of incubation with a multiplicity of infection (MOI) of 10.

### 2.3. Confocal Laser Scanning Microscope (CLSM) Observations

Two batches of samples were prepared for confocal laser scanning microscope observations. All samples were prepared in an Ibidi^®^ brand glass-bottomed µDish©. The first control group contained no virion. It was composed of five samples, one containing 3 mL of M1 culture medium, the second (type A) containing 3 mL of M1 culture and 7.5 µL of PAO1 bacterial solution, and three samples for microscopic observation (composition identical to the control).

The second group was composed of one sample (type B) composed of 3 mL of M1 medium, a sample type A, and three samples that contained a viral load added after 4 h of incubation (37 °C, 50 rpm). They were then incubated again for 20 h after the addition of the bacteriophages. The first virion-free group was directly incubated for 24 h under the same conditions.

When the controls indicated no contamination on agar plates, all samples were treated with 1.2 mL of 70% *v*/*v* formaldehyde for 30 min to fix the biofilm. Excess liquid was then removed with a pipette by suction on the edges of the µDish©.

Bacterial cell labeling was performed by adding 2 µL of Syto9 to the center of the µDish©. The observation parameters were set to an excitation wavelength of 483 nm and an emission wavelength of 505 nm, which are the optimized values. Each of the three samples from the two groups was observed in five different areas. The best shots were selected to illustrate this work and are representative of the entire observed sample.

### 2.4. Radiofrequency Characterization

**Figure 1:**
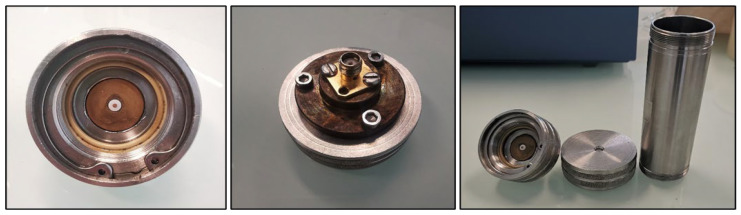
Biofilm sensor pieces. From left to right: coaxial probe top view, coaxial probe bottom view, and the whole sensor detached.

**Figure 2:**
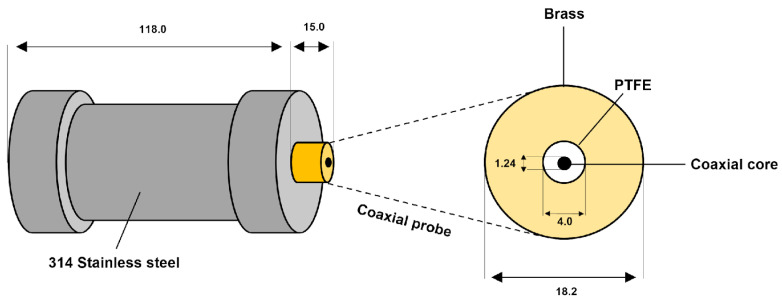
Technical drawing of the sensor mensuration (millimeters) and materials.

In this study, a customized radio frequency sensor derived from the one presented in our previous work [22] was used to investigate the electric properties of biosolutions and run growth kinetics. It is made of a dielectric line wrapped with an insulating layer (PTFE), shown in Figure 1. Thus, when an electromagnetic wave propagates in the coaxial core, an electromagnetic field is formed on the truncated part and radiates in the vicinity of the brass belt. The probe sensing is largely dependent on the solution electroactivity and a reflection coefficient S11 is given as the ratio of the incident to the reflected wave. The diameters of the coaxial core, insulator, and brass cylinder are 1.24, 4.0, and 18.2 mm, respectively (Figure 2). The height of the probe is 15 mm. The 314 stainless steel model reactor was selected due to the need to maintain optimal protection against contamination.

The growth kinetics took place over a minimum of 40 h to allow the bacteria to colonize all the surfaces and establish within a biofilm. Measurements were made every 30 min over the 300 MHz frequency three times using a LabView^®^ (2013) program connected to a vectorial network analyzer Anritsu (France) MS2037C model. A total of 20 mL of sterile M1 culture medium was added to the 314 stainless-steel reservoirs of the sensor (previously sterilized by autoclaving, 121 °C for 30 min). Each growth kinetic was started at the time of inoculation of the system with the PAO1 strain by adding 50 µL of bacterial solution to the tank. The sensor was then closed with its lid and covered with aluminum foil. The whole assemblage was then placed in a thermostatically controlled oven at 37 °C, without agitation. After 4 h, between automatic measurements, the sensor was quickly removed to add 1 mL of viral solution, containing Domino-17 bacteriophage with an estimated MOI of 10 (OD in parallel).

### 2.5. Statistical Analysis

All curves from the radio frequency sensor and optical density represent an average of six successive measurements. The highly variable viral activity within the system does not allow for study reproducibility. The significance of the differences was determined using an analysis of variance (ANOVA). These analyses were programmed and automated on R© software (version 3.6.1). The threshold value of *p* < 0.05 was selected to determine the significance of the observed differences. All tests performed indicate that no statistical difference is present between the 5 successive replicates of each measure.

## 3. Results and Discussion

### 3.1. Optical Density Monitoring

**Figure 3:**
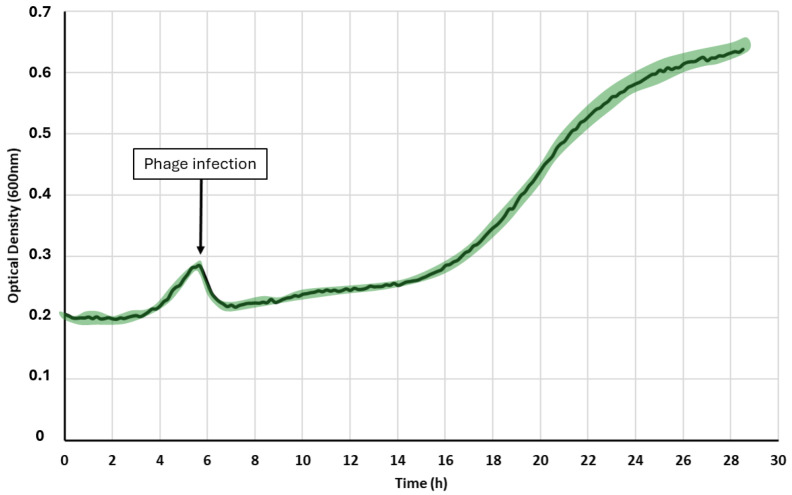
Growth kinetics of *P. aeruginosa* PAO1 following phage injection 6 h after the start of the measurements. The green cloud represents the standard deviation.

Optical density (OD) measurement (around 600 nm) is a method commonly used to monitor bacterial inhibition by phage therapy in a liquid medium [12]. The optical density is used to estimate bacterial concentration. The results of the growth monitoring are presented in Figure 3. The measurement shows a lag phase during the first four hours of monitoring, beyond which the detection limit of the method is reached and the signal begins to increase. The optical density increases from 0.2 to 0.3 within two hours, indicating an increase in the cell density. The injection of the bacteriophages was performed at 6 h. The signal drops immediately but starts to increase again after 8 to 10 h of incubation and then reaches a stationary phase at the end of the kinetics, after 25 h.

Interpretation of these results involves considering several factors that could influence the measurements. Firstly, sedimented cell fragments resulting from viral lysis of bacterial cells may affect the accuracy of the measurements. When bacterial cells are lysed by viruses, the remnants may settle at the bottom of the culture, potentially interfering with optical density readings. In addition, it is not excluded that the bacterium *Pseudomonas aeruginosa* forms a biofilm on the well’s walls. This biofilm formation could alter the distribution of incident light within the culture, leading to a contribution to the recorded optical density that is not solely due to bacterial growth in suspension. Moreover, the extracellular matrix within biofilms has the capacity to absorb a portion of the incident light beam. This absorption can further confound optical density measurements by contributing to the overall recorded value. All these elements combined could potentially bias the monitoring of bacterial growth. However, the crash caused by the addition of viruses cannot be questioned, as cell lysis reduces the content of suspended particles in the culture medium. Thus, we can hypothesize that the strong increase in signal after 10 h of incubation corresponds to the selection and development of bacteria resistant to the injected phages, resulting in an increase in the optical density of the medium. While optical density measurements provide valuable insights into bacterial growth dynamics, they may be influenced by various factors such as biofilm formation, viral lysis, and the presence of sedimented cell fragments. Understanding these potential biases is critical for interpreting experimental results accurately. In the case of a bacterial infection, this bias could have a very strong impact on the diagnostic evolution and the subsequent therapeutic strategy. The virulence of pathogenic bacteria can dramatically increase if the strain switches from a biofilm to a planktonic state [38]. It is now commonly admitted that the biofilm way of life negatively affects the efficiency of biocides and antibiotics [39] and facilitates the adaptation of the strain to treatment by evolutive forcing [40]. Such an incomplete eradication process can lead to a counter-burst infection with a switch from the biofilm strain to planktonic, and so on, ultimately generating, for example, a septic shock [41,42]. This analysis highlights the limits of classical bacterial monitoring performed by optical density. Although straightforward to set up and automatable, this method quickly becomes difficult to use when several micro-organisms and biological phenomena are evolving simultaneously.

### 3.2. Confocal Microscopy Visualization

It remains difficult to estimate the real impact of phages on the growth processes of *P. aeruginosa* without directly observing the behavior of bacteria in their three-dimensional environment. Confocal microscopy allows the realization of a three-dimensional overview of the biofilm architecture and the observation of the impact of bacteriophages on its formation. The following study aims to compare two growth conditions of *P. aeruginosa* bacteria, one under classical growth conditions and the other under predation by the bacteriophage. The biofilm produced by the PAO1 strain, after one day of growth, is mature and covers almost all the immersed surfaces.

**Figure 4:**
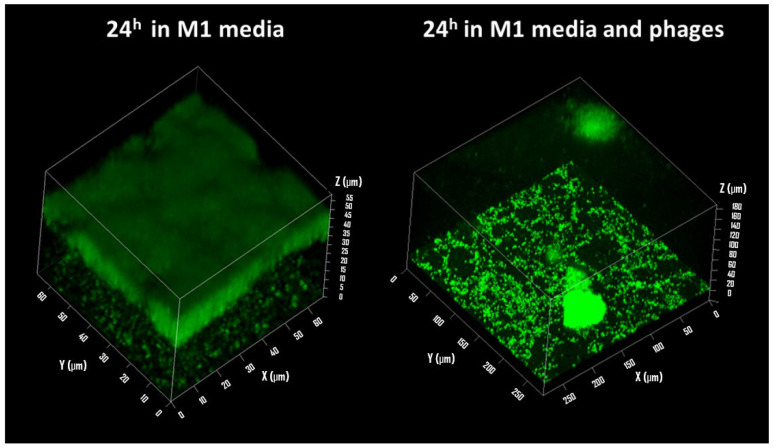
Biofilm of *P. aeruginosa* observed by confocal microscopy after 24 h of growth in M1 media.

Figure 4 displayed a confocal microscope observation of a section of biofilm from a control sample with the aim of assessing the thickness of the bacterial film. In this area, the thickness is between 10 and 20 µm according to the 3D visualization. On the observation of a sample subjected to predation by a bacteriophage, viruses were injected after 4 h of bacterial growth at an estimated MOI of 10 (OD in parallel). In the three-dimensional reconstruction, the bacterial film is extremely thin, and there appears to be only a bacterial monolayer covering the surface. Two isolated aggregates are visible. One of them is located on the surface, partially visible on the upper surface. The second is in contact with the glass surface. In this configuration, it is possible to distinguish some bacteria evolving around this aggregate. The glass surface seems to have been modified, revealing circular areas devoid of chromophore molecules. This can be explained by the death and lysis process within *P. aeruginosa* PAO1 biofilms, which was observed by Webb et al. [43] during bacterial growth in glass flow reactors. Using BacLight LIVE/DEAD viability labeling, it is possible to observe cell death that occurred with a temporal and spatial organization in the biofilm, within microcolonies as circular areas, slightly larger than those observed in Figure 4. After 12 days of biofilm development, up to 50% of the microcolonies in the biofilms showed death and lysis in their centers. Similarly, we observed such a phenomenon in our experiment.

### 3.3. Bacterial Development Monitoring

While numerous studies have emphasized the potential of phages as alternatives to antibiotics and biocides for biofilm treatment, they fall short of fully detailing the dynamics of virus–host interactions. Existing research [44,45,46] typically relies on measurements at daily or hourly intervals, involving labor-intensive cell counts. This approach, constrained by its fixed-time nature, often overlooks critical information in the rapidly evolving host–virus relationship, particularly for fast-metabolizing human pathogenic bacteria.

**Figure 5:**
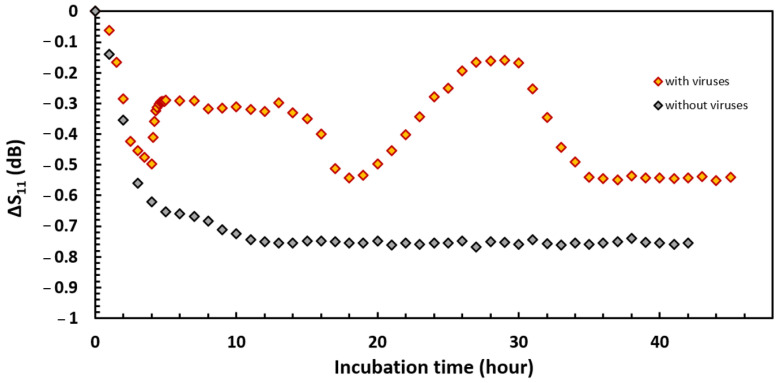
Study of the inhibitory effect of phages on the growth of *P. aeruginosa* performed with the radio frequency sensor, f = 300 MHz.

Our study introduces a novel approach through the application of radio frequency measurements, offering a finer analysis of the dynamic interplay between bacterial populations and their predators/inhibitors. The initial experiment using this method, illustrated in Figure 5, compares the S_11_ coefficient’s variation over time during *P. aeruginosa* growth in the presence of virions against traditional kinetics. The black curve corresponds to the control. The S_11_ coefficient decreases with time and draws two phases corresponding to planktonic growth and biofilm formation as observed in previous experiments with this sensor [22]. This result was expected since the experiment is identical to the previous one, except for the nature of the culture medium [22]. A slope breakpoint is recorded after 8 h of incubation. However, while the total variability of the S_11_ coefficient is only −7.75 dB, the dielectric characterization of *P. aeruginosa* growth in the TSB medium showed a total drop of almost −1.6 dB at the same frequency. This observation provides further evidence that the nature of the culture media plays an important role in the sensor response due to the initial compositional differences.

The orange curve displayed (Figure 5) corresponds to the growth kinetics of the bacteria in the presence of virions. Immediately after the injection of the viruses, the S_11_ coefficient abruptly changes its dynamics and shows an increase of 0.2 dB within a few tens of minutes. A subsequent stationary phase lasting 9 h is established, and then the signal again shows a brief drop immediately followed by a second increase more intense than the first, implying a signal increase of 0.4 dB in 10 h. The S_11_ coefficient then catches up to its lowest level at −0.6 dB and remains stationary until the end of the experiment. Our findings demonstrate a nuanced response of the S_11_ coefficient to biofilm formation and virion interaction, with distinct phases and breakpoints that are not captured in conventional measurement techniques.

The interpretation of these results can be complex, especially when considering the broader ecological implications of phage–bacterial interactions. Like all predators, phages can exert selection pressure and thus lead to the transformation of the bacterial community composition. This phenomenon aligns with the established theory of phage infection, which suggests that phages can drive evolutionary changes within bacterial populations, leading to transformations such as increased drug sensitivity or the emergence of phage-resistant mutants, as evidenced by previous studies [47,48]. These adaptations, including increased extracellular toxin production as noted by Hosseinidoust et al. [49], underscore the complex adaptive dynamics between bacteria and phages. Our method’s ability to detect these nuanced changes in real time offers a significant advancement over the existing literature, which often struggles to pinpoint the specific stages of phage action due to the reliance on time-lapse imaging.

The stages of lysis and bacterial recrudescence do not systematically occur at specific times. The scientific literature on the kinetics of phage action is still poor. Moreover, each type of bacteriophage has different infection and replication rates. As well, bacterial hosts can exhibit various resistance mechanisms [50]. This parameter depends on so many factors that it is currently difficult to provide reliable answers to these two hypotheses, although they are equivalent processes. In our study, irregular viral activity was successfully detected by the proposed radiofrequency method. When biological monitoring is based on images taken at regular intervals in time, it is still difficult to know at which stage of the life cycle the cells are, whereas the sensor provides accurate data in real time. Our findings highlight the great potential of this sensor for the detection of biofilm and the evaluation of the efficiency of anti-biofilm protection.

While our study has provided valuable insights into the dynamics of phage–bacteria interactions within a controlled experimental setting, it is important to recognize that our approach has limitations in terms of its applicability to real-world scenarios. By focusing on monospecies biofilms, we aimed to meticulously investigate specific phage–host interactions, which facilitated a deeper understanding within a controlled environment. However, it is crucial to acknowledge that this simplified model may not fully capture the complexities observed in natural environments and clinical settings, where microbial communities are often diverse and dynamic. Polymicrobial biofilms, in particular, exhibit unique ecological dynamics that may influence their response to phage predation in ways that are not fully represented in our study. Recognizing these limitations emphasizes the importance of future research endeavors to explore phage–bacteria interactions within more ecologically relevant settings, such as multispecies biofilms. By broadening the scope of our investigations, we can enhance our understanding of phage therapy and its potential applications in addressing the complexities of microbial communities encountered in various contexts.

## 4. Conclusions

This work aimed at assessing the efficiency of the newly developed sensor for providing new insights into bacterial colonization and biofilm formation during bacteriophage predation using *P. aeruginosa* in M1 media. Conventional methods, such as optical density kinetics and CLSM observations, have highlighted the lack of temporal information that hides important phenomena. Our research reveals the sensor’s capability to detect irregular viral activity, a feature not adequately addressed in the current scientific literature. Traditional biological monitoring methods based on interval-based imaging fall short of providing real-time, stage-specific information about the cells. In contrast, our sensor delivers precise, real-time data, thereby significantly enhancing our understanding of biofilm dynamics and the effectiveness of anti-biofilm strategies. The sensor avoids the measurement drawbacks of microscopic and optical density techniques. However, it requires a good knowledge of the model bacteria studied and it is difficult to use it alone without the help of standard microbiology methods, like optical density and fluorescence tests. Therefore, we propose to use this sensor as a support tool to complement the existing methods. These results open the discussion on the optimal use of the sensor to clarify the biological process that occurs during biofilm formation. The use of this type of sensor could be crucial for further bacterial studies, especially prey/predator dynamics (e.g., phage cocktail, *Bdellovibrio* predation), and can potentially improve actual models. In conclusion, our study not only bridges the gaps identified in the existing literature regarding the real-time monitoring of virus–host dynamics but also paves the way for more advanced and accurate methods of understanding and combating bacterial biofilms.

## Data Availability

Data are openly available in a public repository.
